# Quantifying hydration-related measures and their association with wearable-derived ergonomic risk in warehouse workers

**DOI:** 10.3389/fpubh.2026.1903472

**Published:** 2026-08-12

**Authors:** Philip J. Agostinelli, Michal Ozga, Cole Murphy, Zadok Isaacs, Peter John D. De Chavez, Liam R. Brown, Alyssa Annarelli, Jon K. Davis

**Affiliations:** 1 Gatorade Sports Science Institute, Life Sciences, PepsiCo R&D, Bradenton, FL, United States; 2 Gatorade Sports Science Institute, Life Sciences, PepsiCo R&D, Valhalla, NY, United States; 3 Gatorade Sports Science Institute, Life Sciences, PepsiCo R&D, Frisco, TX, United States; 4 Data Science & Analytics, PepsiCo Global R&D, Plano, TX, United States; 5 Gatorade Sports Science Institute, Life Sciences, PepsiCo R&D, Leicester, United Kingdom

**Keywords:** manual labor, manual material handling, occupational stress, physically demanding work, physiological monitoring, wearable sweat sensors

## Abstract

**Introduction:**

Warehouse workers may experience prolonged heat exposure and sweat loss, which can influence fatigue and movement patterns related to ergonomic risk. However, evidence in this occupational group remains limited. Therefore, we aimed to quantify acute (pre- and post-shift) and across-week (Days 1–4) changes in urine specific gravity (USG) and explore the associations between hydration-related measures (*Δ* urine color, sweat rate, and total fluid intake), wearable-derived ergonomic risk metrics, and self-reported fatigue.

**Methods:**

A total of 25 employees at a Florida beverage bottling facility completed pre- and post-shift urine sampling over four workdays, recorded total fluid intake, and self-reported fatigue/energy. Sweat volume, sweat rate, sweat sodium concentration, and ergonomic risk metrics were measured on Days 2–4 using wearable devices. Linear mixed-effects models were used to assess USG changes by time point and day. Multiple linear regression was used to examine the associations between predictors (*Δ* urine color, sweat rate, and total fluid intake) and ergonomic risk outcomes and pre-to-post-shift changes in fatigue/energy.

**Results:**

USG increased from pre- to post-shift (*p* = 0.03), with no significant effect of day across the workweek (*p* = 0.58). Change in urine color was significantly associated with the injury risk score (*p* = 0.03), total elevated risk movement count (*p* < 0.01), and mild-risk movement count (*p* < 0.01). Sweat rate was a significant predictor of changes in physical fatigue from pre- to post-shift (*p* = 0.03). Night-shift workers demonstrated higher activity scores and sweat sodium loss than morning-shift workers (*p* < 0.01).

**Conclusion:**

Workers frequently presented with concentrated urine both pre- and post-shift, and a greater within-shift worsening of urine color was associated with a higher occurrence of certain wearable-derived ergonomic risk movements.

## 1 Introduction

Working in warehouse facilities requires high levels of energy expenditure, mental acuity, and technical skills, sometimes in hot and humid environments. Previous data indicate that individuals in various physically demanding occupations experience average whole-body sweating rates (WBSR) of between 0.3 and 1.5 L/h (1–4), similar to sport athletes (5). Prior research also reports that under hot and humid environmental conditions, dehydration leads to an increased risk of heat-related illness and reductions in cognitive function, alertness (6, 7), motor control (8), and physical work capacity and productivity (9, 10). These impairments may increase a worker’s likelihood of ergonomic movement errors during manual tasks (11). However, the majority of research on hydration and heat strain in physically demanding work has focused on firefighters (12), military personnel (13), and outdoor laborers (3, 4, 14–15), leaving workers engaged in manual tasks, such as warehouse employees, underrepresented, as highlighted by a recent scoping review (16). This absence of literature is increasingly pressing when considering the occupational burden of heat stress, as Occupational Safety and Health Administration (OSHA) data suggest that this is a prevalent issue in occupational sectors, with 85% of reported exertion injuries related to heat stress (17).

Currently, the National Institute for Occupational Safety and Health (NIOSH) recommends consuming 250 mL of water or other fluids every 15–20 min during physically demanding work or, in the case of prolonged exposure (> 2 h) in hot environments, recommends sports drinks containing <8% carbohydrates and electrolytes (9). These general guidelines provide a starting point; however, more personalized recommendations may promote better hydration habits given the diverse physiological demands of industrial athletes. Some research in a laboratory setting has shown that ad libitum drinking under various environmental conditions and work rates may prevent clinically meaningful dehydration (18), while other field-based literature suggests that these recommendations may be insufficient in practice because of their effectiveness and difficulties with implementation, as studies have shown that over 80% of agricultural workers were underhydrated at the end of their shift (urine specific gravity (USG) > 1.020) (15).

Additionally, although these guidelines are designed to prevent the onset of dehydration while on the job, they do not account for workers who begin their shifts in an underhydrated state. This distinction is important, as data suggest that about 50% of agricultural workers arrive at work underhydrated (15). Specifically, the associations of markers of hydration status and sweat profiles with ergonomic injury risk in warehouse workers remain unclear. Therefore, the primary aim of this study was to assess both acute and weekly changes in pre- and post-shift USG in warehouse workers at a beverage bottling facility during a typical workweek. Second, we examined the extent to which urine color, fluid intake, and sweat rate were associated with wearable-derived ergonomic risk, self-reported fatigue, and self-reported energy. Finally, we explored and quantified potential differences in these metrics between those who worked morning shifts versus night shifts. We hypothesized that USG would increase from pre- to post-shift due to insufficient fluid intake during work. We also hypothesized that USG would increase over the course of the workweek owing to the compounding effect of insufficient daily fluid intake at work. Additionally, we hypothesized that elevated urine color and lower fluid intake values, paired with higher sweat losses, would be associated with a higher occurrence of elevated ergonomic risk.

## 2 Materials and methods

### 2.1 Participants

A total of 30 individuals who worked in picker and loader roles at a local beverage bottling facility in Florida were enrolled in the study. Participants met the following inclusion criteria: (i) self-identified as healthy; (ii) between the ages of 18 and 65 years; (iii) currently employed at the bottling facility site; (iv) able to read and comprehend English; (v) employed for 2 months or longer; and (vi) willing to adhere to all study procedures outlined in the informed consent form. Participants were excluded from the study if (i) they were allergic to adhesives or (ii) they were currently pregnant or nursing. Participants were withdrawn if they could not complete all four sessions within the 1-week testing period. However, partial data were not retained for applicable analyses if participants completed more than one trial. This study was approved by the Sterling Institutional Review Board (Atlanta, GA, United States) (protocol number: 13999; approved: 25 June 2025) and was conducted in accordance with the ethical standards of the Declaration of Helsinki. This study was registered with ClinicalTrials.gov (NCT07117773) on 12 August 2025.

### 2.2 Study design

#### 2.2.1 Study overview

This longitudinal observational study involved five visits. The first visit involved participant screening and consent at least 2 weeks before the start of the data collection. After providing informed consent, the participants were asked to complete a general health questionnaire. Participants then completed pre-, during, and post-shift assessments at the bottling facility for 4 days during a typical workweek (Days 1–4). Operational leadership at the bottling facility provided time and space for recruitment and data collection; however, they were not involved in any study procedures and did not have access to the study data. Statistical analysis was conducted by PJA and PJDD. The authors have retained full control over data analysis, interpretation, and the decision to submit the manuscript for publication.

#### 2.2.2 Baseline measurements (Day 1 only)

Before the start of the shift on Day 1, participants were asked to complete a series of baseline surveys using EyeQuestion^®^ (Elst, Netherlands) on their personal smartphones or on a smartphone provided by the study team. These baseline surveys included the following: (1) Pittsburgh Sleep Quality Inventory; (2) Rapid Eating Assessments for Subjects—Short Form; (3) Global Physical Activity Questionnaire—Short Form; (4) Hydration Habits and Knowledge Questionnaire; and (5) Demographic Questionnaire. However, only the demographic questionnaire responses were analyzed in this study. After completing these questionnaires, height and body mass were measured using a Tanita WB-800S Plus digital weight scale (Tanita^®^, Tokyo, Japan), with the participants in a full uniform (clothing and shoes). These measurements were completed only on the first day of data collection.

#### 2.2.3 Pre-shift measurements (Days 1–4)

Participants were asked to complete a survey on their phone via EyeQuestion regarding the prior night’s sleep and a 100-mm visual analog scale (VAS) involving questions about their current feelings of mental energy, mental fatigue, physical energy, physical fatigue, sleep quality, sleep quantity, and thirst sensation. Participants were then asked to provide a urine sample to assess their hydration status, including USG and urine color. USG was measured using a PEN-Urine S.G.^®^ digital handheld refractometer (Atago Co., Ltd., Tokyo, Japan). The refractometer was calibrated in distilled water before analyzing any samples for each assessment period, including the pre- and post-shift time points. USG was measured immediately after sample collection, in duplicate. If the measurements were not identical, a third measurement was taken, and the average of the three values was used. Urine color was measured using a 1–7 scale (19, 20) and rated by the same study team member under consistent lighting conditions.

During Days 2, 3, and 4, participants were set up with Epicore^®^ Connected Hydration (Cambridge, MA, United States) and LifeBooster^®^ Senz (Vancouver, Canada) devices to monitor sweat-related outcomes and ergonomics, respectively. The Epicore^®^ Connected Hydration device was placed on the skin of the lateral portion of the dominant upper arm, parallel to the humerus, using an adhesive patch. The LifeBooster Senz devices were placed on the right and left forearms and upper arms, as well as on the pelvis and thorax. The forearm bands were securely placed approximately 1–2 inches above the wrist using a Velcro band. The upper arm sensor was securely placed approximately 2–3 inches below the head of the humerus using a Velcro band. The placement was verified to ensure that no interference occurred with the placement of the Epicore^®^ Connected Hydration Device. The pelvic sensor was securely placed on the left side of the waist using a pants waist clip. The final sensor was securely placed on the back of the participant’s shirt collar, below the base of the neck. Participants wore fitted shirts to ensure that sensor placement was maintained throughout their shift. After being fitted with both wearable devices, the Connected Hydration device was paired to a smartphone, and LifeBooster sensor orientation calibration was performed. Following calibration, the participants proceeded to start their shift.

#### 2.2.4 During shift measurements

During their shift (Days 1–4), the investigative team monitored fluid intake. To measure fluid intake, participants provided each beverage to the study team before drinking it to obtain a pre-beverage mass. This value was recorded by the study team to the nearest 0.001 kg using a Compass^™^ CR electronic scale (Ohaus, Parsippany, New Jersey, United States). When the participants finished each beverage, they returned the bottle to the study team to record the post-beverage mass. Beverage type was recorded and classified as water, sports drink, energy drink, soft drink, coffee, juice, or other. This was repeated for all fluids consumed by the participants during the entire work shift to accurately track the total fluid intake. Temperature, humidity, and wet-bulb globe temperature (WBGT) were measured throughout the facility in the primary work areas at four evenly spaced time points throughout the course of each shift using a Kestrel 5,400 (Nielsen-Kellerman^®^, Boothwyn, Pennsylvania, United States). Additionally, throughout the participant’s shift, the study team periodically verified that Epicore^®^ and LifeBooster sensor placement was maintained.

#### 2.2.5 Post-shift measurements

Approximately 15 min before the end of their shift, participants were asked to complete the same VAS surveys conducted before the shift surveys related to mental and physical energy, fatigue, and current sensations of thirst. Following survey completion, participants removed the Epicore^®^ Connected Hydration and LifeBooster^®^ Senz devices. Finally, the participants provided another urine sample before leaving the facility.

### 2.3 Survey outcomes

#### 2.3.1 State mental and physical energy and fatigue visual analog scale

The state aspect of the Mental and Physical State and Trait Energy and Fatigue Scale (21) was used to assess participants’ feelings of mental energy, mental fatigue, physical energy, and physical fatigue at that moment. This portion of the survey consisted of 12 items using a 0–100-mm VAS. Each item was anchored on the left by an “absence of feeling” (0) and on the right by the “strongest intensity of feeling” (100). Participants were asked to move the vertical line across the horizontal line to indicate the intensity of their feelings at that moment. Scores were automatically generated using EyeQuestion. Cronbach’s *α* values between 0.71 and 0.94 have been observed for state mental and physical energy and fatigue (22, 23). Of the 12 items, each construct (physical energy, physical fatigue, mental energy, mental fatigue) was assessed using 3 related questions, which were averaged to obtain 1 score per construct. Physical and mental energy each included feelings of energy, vigor, and pep. Physical and mental fatigue each included feelings of fatigue, exhaustion, and being worn out. Change scores from pre- to post-shift were used in our secondary aim to account for the level of fatigue the participants were already experiencing upon arrival at work.

#### 2.3.2 Thirst visual analog scale

Participants then proceeded to complete a thirst assessment in EyeQuestion, completing it using the thirst sensation scales (TSS). The TSS consists of 6 questions using a 0–100-mm VAS. This survey was chosen based on its usage in previous hydration and exercise research (24, 25). Questions included “How thirsty do you feel right now?” (thirst), “How pleasant would it be to drink some water right now?” (pleasantness to drink), “How dry does your mouth feel right now?” (mouth dryness), “How would you describe the taste in your mouth right now?” (mouth taste), “How full does your stomach feel right now?” (stomach fullness), and “How sick to your stomach do you feel right now?” (stomach sickness). Each question was anchored on the left by “not at all” and on the right by “very.” TSS scores were used to quantify the average level of thirst perceived by warehouse workers before arrival at and departure from work.

#### 2.3.3 Sleep quality and quantity questions

Participants then completed a series of four questions related to their previous night’s sleep. These questions included the time they went to bed the night before, the time it took them to fall asleep, total hours of sleep, and perception of sleep quality. Sleep quality consisted of a 0–100-mm VAS. The sleep quality VAS asked, “How would you rate the quality of your sleep last night?” and was anchored on the left by “worst” and on the right by “best.” Sleep questionnaire responses were used to quantify the warehouse workers’ average self-reported sleep quantity and quality before their shifts.

### 2.4 Wearable device outcomes

#### 2.4.1 Epicore^®^ connected hydration outcomes

The Epicore^®^ Connected Hydration Monitor is a wearable microfluidic biosensor that allows continuous monitoring of sweat and sweat sodium losses and is specifically designed for use in occupational settings. This system has shown strong agreement (R^2^ ≥ 0.70) with reference methods for regional sweat collection (26) while allowing for continuous monitoring throughout the full duration of the participant’s shift. Key metrics from the Connected Hydration device included total sweat loss, sweat rate (total sweat loss divided by shift duration, in L/h), total sweat sodium loss, sweat sodium concentration (mmol/L), activity level, and skin temperature. The sampling rate for sweat-related measurements was 0.05 Hz (1 sample every 20 s). Activity level was calculated using triaxial accelerometry. Accelerometry values are then aggregated and rescaled using a proprietary vendor-derived algorithm to an approximate 20–40-unit range. To understand the scaling, an increase in this activity score from 28 to 29 would indicate a doubling of total acceleration experienced during a 5-min period. Small movements during sedentary activity, such as office work, produce scores in the upper 20s; activities such as walking on a treadmill produce scores in the 30–35 range; and running produces scores that can reach 40. Accelerometry and skin temperature data were sampled at 25 Hz and then aggregated to provide 1-min values. The total sweat loss and sweat sodium loss were averaged across the three observation days. Average sweat sodium concentrations, activity levels, and skin temperatures were calculated for each shift and then averaged across the three observation days to provide 1 average value for each metric.

#### 2.4.2 LifeBooster Senz outcomes

**Figure 1:**
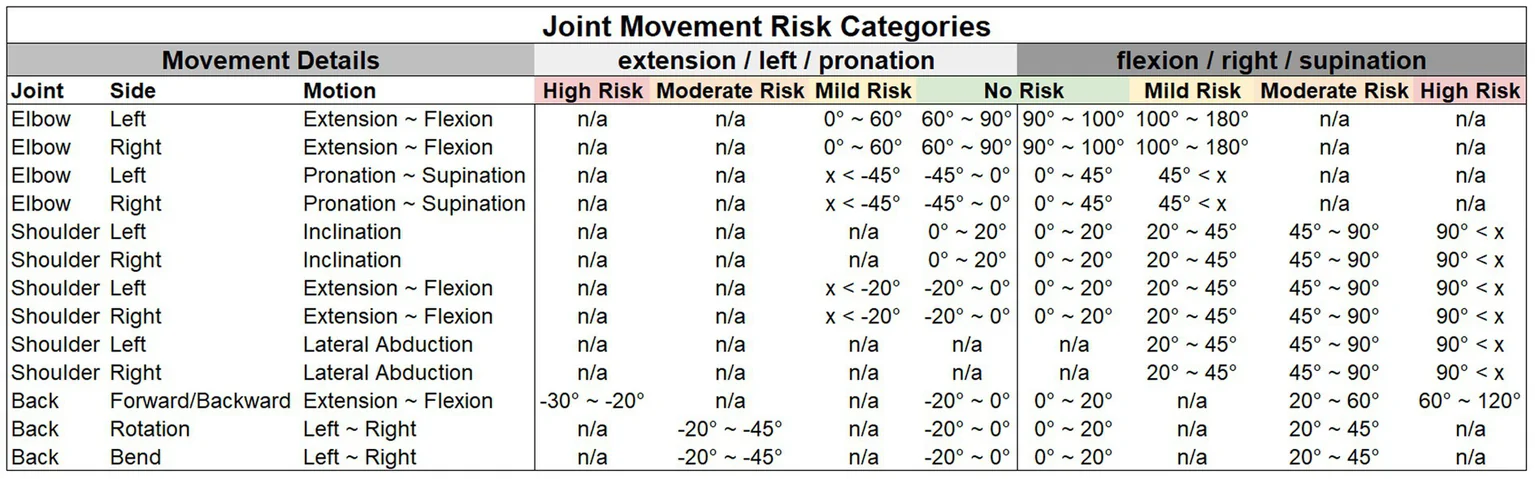
Joint movement risk classifications based on joint, side, motion, and position relative to the body.

The LifeBooster Senz sensors include six triaxial accelerometers (right upper arm, right forearm, left upper arm, left forearm, pelvis, and thorax) that pair with the LifeBooster Senz platform to provide a comprehensive analysis of ergonomic risk among workers. The sampling rate of the LifeBooster Senz sensors was 20 Hz (1 sample every 50 ms). Total values for the entire shift were used and then averaged to obtain an average value for each outcome variable across the three observation days. Five key ergonomic outcome variables were derived from the LifeBooster Senz for this investigation: (1) injury risk score, (2) total elevated risk movement count, (3) high-risk movement count, (4) moderate-risk movement count, and (5) mild-risk movement count. The injury risk score was quantified using the SenzOne system (LifeBooster Inc., Canada), a proprietary composite risk metric ranging from 0–100, where higher values indicate greater cumulative musculoskeletal injury risk. The score integrates multiple ergonomic risk domains, including repetitive strain, sustained posture, lifting mechanics, hand-arm vibration, and heat stress across key body regions (hands, elbows, shoulders, back, and core). Each domain is derived from established ergonomic standards, including the WorkSafeBC C4-2 directive (repetitive strain) (27), Rapid Upper Limb Assessment (RULA; adapted posture scoring) (28), ISO-5349 (vibration exposure) (29, 30), the NIOSH Variable Lifting Index (lifting) (31, 32), and American Conference of Governmental Industrial Hygienists (ACGIH) Threshold Limit Values (heat stress) (33). Individual risk components are aggregated using a probabilistic union function, which estimates the likelihood of at least one injury-related exposure occurring across all measured domains. This approach allows for fractional accumulation of risk while bounding the score asymptotically at 100. Total elevated risk movement count represents the number of times a participant experienced a movement pattern classified as posing an elevated risk of ergonomic injury. This measure is the sum of high-risk, moderate-risk, and mild-risk movement counts. The criteria used to develop high-risk, moderate-risk, and mild-risk movement categories were adapted from the RULA (28), and the classifications for each category are shown in Figure 1.

### 2.5 Statistical analysis

In accordance with our primary aim, a linear mixed-effects model using time point (pre- and post-shift), Days 1–4, and their interaction was specified as fixed effects to examine acute and weekly changes in USG throughout the workweek. Participant ID was specified as a random intercept to account for the repeated measurements. In the event of significant (*p* ≤ 0.05) main or interaction effects, post-hoc comparisons were conducted using Tukey-adjusted pairwise comparisons. This model was fit using maximum likelihood, which allows participants with incomplete repeated measures to contribute available data under a missing-at-random assumption. In accordance with our secondary aim, a multiple linear regression model was used to explore associations between predictors (*Δ* urine color, sweat rate, and total fluid intake) from Days 2–4 and the outcome variables (injury risk score, total elevated risk movement count, high-risk movement count, moderate-risk movement count, and mild-risk movement count) from Days 2–4. The same model was used to explore associations with average weekly pre- to post-shift changes in physical energy, physical fatigue, mental energy, and mental fatigue. Given the number of secondary regression models evaluated across multiple related outcome variables, these analyses were considered exploratory. As such, no formal adjustment for multiple comparisons was applied, and therefore, results are interpreted with caution in the context of increased family-wise error risk. Urine color was specifically chosen over USG when looking at factors that are associated with ergonomic risk, as it is more practical for use by employees and environmental health and safety staff. This decision was made *a priori*; however, during analysis, to confirm that the use of urine color in place of USG was appropriate, a repeated-measures correlation (34) was used to assess within-participant associations between urine color and USG across all sampling time points. All predictors and outcomes represented weekly averages for each participant. Participants (*n* = 2) with missing wearable data due to device reading issues were removed from this portion of the analysis. All predictors were included in the model simultaneously. Effect sizes were derived from the general linear model framework and expressed as semi-partial R^2^ to quantify the proportion of model-explained variance attributable to each predictor. Independent *t-*tests were performed for sweat, fluid, and activity metrics between participants who worked night shifts and morning shifts to assess potential differences by shift format. As these were different workers on unique shift schedules, age, body mass index (BMI), and shift duration were assessed using independent *t-*tests to evaluate for potential confounding factors. All data were assessed for normality using the Shapiro–Wilk test and quantile-quantile (Q-Q) plot inspection. Homoscedasticity was assessed using scatter plots of the residuals. Variance inflation factor (VIF) values and correlation coefficients of the predictor variables were used to assess multicollinearity. VIF values were all less than 3.0 (35). Data are expressed as mean ± standard deviation. Statistical significance was set *a priori* at a *p*-value of ≤ 0.05 for all analyses. G*Power software was used a priori to determine that a sample size of 25 total participants was required to adequately power the study (*α* = 0.05; 1 – *β* = 0.80; *d* = 0.59). The estimated effect size was derived from results obtained during pilot testing of our main outcome (pre- and post-shift USG) in a similar bottling facility, followed by adjustments for larger expected differences due to elevated environmental and occupational demands at the factory used in this investigation. Statistical analyses were conducted using R Statistical Programming Software Version 4.5.1 (RStudio, Boston, MA, United States).

## 3 Results

### 3.1 Demographic characteristics

Of the 30 participants enrolled, 5 participants withdrew due to scheduling conflicts, leaving a total of 25 participants. Sample size varied by measure due to missing Connected Hydration data. Of those 25 participants, 36% (9) identified as “Black/African American,” 36% (9) identified as “Other,” 12% (3) identified as “Native American,” 8% (2) identified as “White,” and 8% (2) chose not to disclose their race. Additionally, 60% (15) reported their ethnicity as “Hispanic,” 24% (6) reported their ethnicity as “non-Hispanic,” and 16% (4) chose not to disclose their ethnicity. The average environmental conditions experienced by the workers were as follows: WBGT: 27.6 ± 0.8 °C; Temp: 30.5 ± 0.7 °C; and relative humidity (RH): 75.3 ± 2.0. Workers’ shifts lasted an average of 9.0 ± 0.7 h. Mean urine, sweat, total fluid intake, and ergonomic risk metrics are presented in Table 1. Survey data including energy, fatigue, sleep, and thirst metrics are presented in Table 2. Mean fluid intake by beverage types is presented in Table 3.

**Table 1:** Weekly averages for sweat, fluid intake, urine, and ergonomics.

Variable	*n*	Mean ± SD	Min	Max
USG	25	Pre: 1.022 ± 0.005 Post: 1.024 ± 0.006	Pre: 1.009 Post: 1.009	Pre: 1.031 Post: 1.033
Urine color	25	Pre: 4.2 ± 0.9 Post: 4.6 ± 0.9	Pre: 2.5 Post: 3.0	Pre: 6.3 Post: 7.0
Total fluid intake	25	2,774 ± 667 g	1785 g	4,428 g
Total sweat loss	23	3,754 ± 2,627 mL	1,020 mL	10,144 mL
Sweat rate	23	0.41 ± 0.26 L/h	0.11 L/h	1.14 L/h
Total sweat sodium loss	23	3,599 ± 2,627 mg	756 mg	9,250 mg
Sweat sodium concentration	23	34.9 ± 12.8 mmol/L	19.0 mmol/L	59.3 mmol/L
Activity level	24	32.76 ± 1.17 units	30.76 units	34.83 units
Skin temperature	24	33.0 ± 0.5°C	31.9°C	33.7 °C
Total elevated movement count	25	56,149 ± 10,009	36,884	79,924
High-risk movements	25	6,076 ± 1957	2,813	11,120
Moderate-risk movements	25	21,312 ± 3,992	15,370	30,543
Mild-risk movements	25	28,761 ± 6,683	14,055	42,330
Injury risk score	25	87.4 ± 5.0	76.5	95.8

These values represent averages across the workweek; USG: urine specific gravity; Pre = before-shift measurement; Post = end-of-shift measurement.

**Table 2:** Weekly averages for survey responses.

Variable	*n*	Mean ± SD	Min – Max
Physical energy	25	Pre: 44.5 ± 22.4 Post: 40.9 ± 20.8	Pre: 0.0–97.9 Post: 0.0–82.0
Physical fatigue	25	Pre: 26.6 ± 19.4 Post: 36.7 ± 21.1	Pre: 0.0–98.2 Post: 0.0–82.0
Mental Energy	25	Pre: 42.8 ± 22.8 Post: 39.3 ± 22.3	Pre: 0.0–96.8 Post: 0.00–97.7
Mental fatigue	25	Pre: 27.3 ± 20.0 Post: 35.3 ± 23.5	Pre: 0.00–62.8 Post: 0.0–88.0
Thirst	25	Pre: 29.5 ± 18.9 Post: 34.9 ± 23.1	Pre: 0.0–79.3 Post: 0.0–79.4
Pleasantness	25	Pre: 47.2 ± 19.6 Post: 42.8 ± 18.0	Pre: 0.0–80.4 Post: 0.0–74.4
Mouth taste	25	Pre: 48.8 ± 24.9 Post: 47.3 ± 19.6	Pre: 0.0–97.2 Post: 0.0–97.3
Mouth dryness	25	Pre: 27.3 ± 18.4 Post: 26.4 ± 20.7	Pre: 0.0–65.0 Post: 0.0–60.9
Stomach fullness	25	Pre: 26.3 ± 20.4 Post: 29.3 ± 18.2	Pre: 0.0–66.8 Post: 0.0–68.7
Sickness	25	Pre: 16.8 ± 18.4 Post: 19.5 ± 19.4	Pre: 0.0–57.8 Post: 0.0–68.7
Sleep quality	25	Pre: 49.8 ± 17.8	Pre: 0.0–98.1
Hours of sleep	25	6.2 ± 1.2 h	4.3–8.8 h

All items except hours of sleep represent a VAS score from 0–100. These values represent averages across the workweek (days 1–4); Pre = before-shift measurement; Post = end-of-shift measurement.

**Table 3:** Weekly averages for fluid intake types.

Beverage type	*n*	Mean ± SD	Min – Max
Water	25	2,301 ± 880 g	503–4,878 g
Sports Drink	25	296 ± 400 g	0–1,435 g
Energy Drink	25	28 ± 112 g	0–708 g
Soft Drink	25	95 ± 224 g	0–978 g
Coffee	25	54 ± 145 g	0–745 g
Juice	25	24 ± 119 g	0–698 g
Other	25	201 ± 315 g	0–1,055 g

Data reported as mean ± standard deviation.

### 3.2 Urine specific gravity

**Figure 2:**
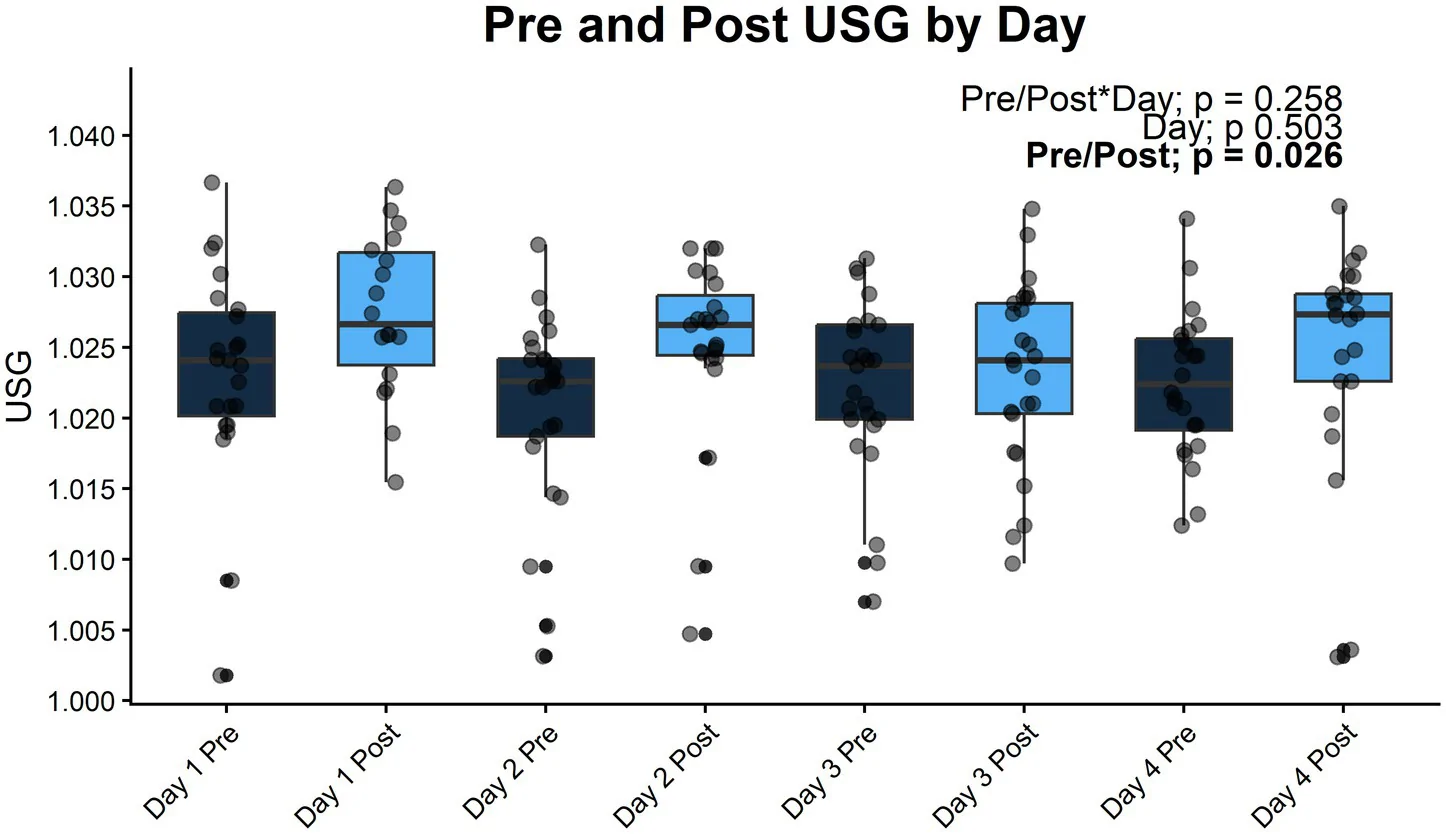
Urine specific gravity (USG) by time point (pre- and post-shift) and day (Days 1–4); *n* = 25 participants.

A linear mixed-effects model revealed a significant main effect of time point (pre- and post-shift) on USG (*β* = 0.0041, SE = 0.0019, t = 2.14, *p* = 0.034, 95% CI [0.0003, 0.0079]). However, there was no significant main effect of day on USG (β = 0.0005, SE = 0.0011, t = 0.56, *p* = 0.576, 95% CI [−0.0015, 0.0027]). There was no significant interaction between time point and day USG (β = −0.0007, SE = 0.0007, t = − 1.01, *p* = 0.258, 95% CI [−0.0021, 0.0007]). A visual representation of these data is shown in Figure 2.

### 3.3 Hydration and ergonomics

To confirm that the use of urine color in place of USG was appropriate, a repeated-measures correlation analysis was performed between the two variables. This analysis confirmed there was a strong, significant repeated-measures correlation (r_rm_ = 0.80; *p* < 0.001). A multiple linear regression model was used to examine whether change in urine color, sweat rate, and total fluid intake were associated with injury risk score. The overall model was not significant [F(3,19) = 2.50, *p* = 0.090, R^2^ = 0.28, adj R^2^ = 0.17]; however, urine color was a significant individual predictor (t = 2.37; *p* = 0.03; semi-partial R^2^ = 0.23, 95% CI [0.3, 5.1]). A multiple linear regression model was then used to examine whether change in urine color, sweat rate, and total fluid intake were associated with total elevated risk movement count. The overall model was significant [F(3,19) = 3.87, *p* = 0.02], explaining 28% of the variance in total elevated ergonomic risk (R^2^ = 0.38; adj R^2^ = 0.28). Individual predictors were examined further and indicated that the change in urine color was a significant predictor (t = 3.23; *p* = 0.004; semi-partial R^2^ = 0.35, 95% CI [2439.3, 11405.9]), but not total fluid intake (t = 0.37; *p* = 0.71; semi-partial R^2^ < 0.01, 95% CI [−4.8, 6.8]) or sweat rate (t = 0.25; *p* = 0.81; semi-partial R^2^ < 0.01, 95% CI [−13308.9, 16856.0]). A multiple linear regression model was then used to examine whether change in urine color, sweat rate, and total fluid intake were associated with total high-risk ergonomic movement count. The overall model was not significant [F(3,19) = 2.19, *p* = 0.122, R^2^ = 0.26; adj R^2^ = 0.14]. A multiple linear regression model was used to examine whether change in urine color, sweat rate, and total fluid intake were associated with moderate-risk ergonomic movement count. The overall model was not significant [F(3,19) = 2.03, *p* = 0.144, R^2^ = 0.24; adj R^2^ = 0.12]. A multiple linear regression model was used to examine whether change in urine color, sweat rate, and total fluid intake were associated with mild-risk ergonomic movement count. The overall model was significant [F(3,19) = 3.47, *p* = 0.037], explaining 25% of the variance in mild-risk ergonomic movement count (R^2^ = 0.35; adj R^2^ = 0.25). Individual predictors were examined further and indicated that change in urine color was a significant predictor (t = 3.21; *p* = 0.005; semi-partial R^2^ = 0.35, 95% CI [1634.7, 7740.4]), but not total fluid intake (t = −0.19; *p* = 0.853; semi-partial R^2^ < 0.01, 95% CI [−4.3, 3.6]) or sweat rate (t = −0.82; *p* = 0.425; semi-partial R^2^ = 0.03, 95% CI [−14269.6, 6270.9]). See Table 4 for full model details.

**Table 4:** Model prediction for hydration and ergonomics.

	Intercept		Total fluid intake		Δ Urine color		Sweat rate	
Variable	Estimate	Std. error	Estimate	Std. error	Estimate	Std. error	Estimate	Std. error
Injury risk score	82.9	4.7	9.0 × 10^−4^	1.4 × 10^−3^	2.7	1.1	2.5	3.8
Total elevated risk movement count	50623.6	8887.8	1.0	2.8	6922.6	2142.0	1773.5	7206.1
High-risk movements	2031.63	1900.1	1.1	0.6	478.4	457.9	1891.7	1540.6
Moderate-risk movements	18400.0	3914.0	0.3	1.2	1757.0	943.3	3881.0	3173.0
Mild-risk movements	30189.6	6053.0	−0.36	1.9	4687.5	1458.6	−3999.4	4906.9

Estimates are unstandardized regression coefficients (β) ± standard error (SE); Δ = change from pre- to post-shift; *n* = 23 participants.

### 3.4 Hydration and self-reported energy and fatigue

A multiple linear regression model was used to examine whether change in urine color, sweat rate, and total fluid intake were associated with changes in self-reported physical energy. The overall model was not significant [F(3,19) = 0.63, *p* = 0.604, R^2^ = 0.09, adj R^2^ = −0.05]. A multiple linear regression model was then used to examine whether change in urine color, sweat rate, and total fluid intake were associated with changes in self-reported physical fatigue. The overall model was not significant [F(3,19) = 2.03, *p* = 0.144]; however, sweat rate was a significant individual predictor (t = 2.30; *p* = 0.033; semi-partial R^2^ = 0.09, 95% CI [2.3, 49.9]). A multiple linear regression model was used to examine whether change in urine color, sweat rate, and total fluid intake were associated with changes in self-reported mental energy. The overall model was not significant [F(3,19) = 1.37, *p* = 0.282, R^2^ = 0.18, adj R^2^ = 0.05]. A multiple linear regression model was then used to examine whether change in urine color, sweat rate, and total fluid intake were associated with changes in self-reported mental fatigue. The overall model was not significant [F(3,19) = 1.14, *p* = 0.358, R^2^ = 0.15, adj R^2^ = 0.02]. See Table 5 for full model details.

**Table 5:** Model prediction for hydration and changes in self-reported energy and fatigue.

	Intercept		Total Fluid Intake		Δ Urine Color		Sweat Rate	
Variable	Estimate	Std. error	Estimate	Std. error	Estimate	Std. error	Estimate	Std. error
Δ Physical energy	−11.7	11.7	4.0 × 10^−3^	4.0 × 10^−3^	−1.54	2.82	6.5	9.5
Δ Physical fatigue	−22.2	14.0	1.0 × 10^−3^	4.0 × 10^−3^	−4.4	3.4	26.1	11.4
Δ Mental energy	−15.8	11.1	6.0 × 10^−3^	3.0 × 10^−3^	−2.2	2.7	2.2	9.0
Δ Mental fatigue	−13.3	14.0	7.0 × 10^−4^	4.0 × 10^−3^	−5.3	3.4	14.1	11.4

Estimates are unstandardized regression coefficients (β) ± standard error (SE); Δ = change from pre- to post-shift; *n* = 23 participants.

### 3.5 Occupational demands by shift

Environmental conditions were quantified for both morning-shift (Temp 30.9 °C, WBGT 28.5 °C, RH 76.7%) and night-shift workers (Temp 29.5 °C, WBGT 26.4 °C, RH 72.4%). BMI and age were not significantly different between morning- and night-shift workers (t = 1.14, *p* = 0.267, d = −0.5, 95% CI [−1.70, 5.82]; t = 1.80, *p* = 0.085, d = −0.78, 95% CI [−0.88, 12.75], respectively). Shift duration was not significantly different between morning- and night-shift workers (t = −1.85, *p* = 0.078, d = 0.83, 95% CI [−0.82, 0.05]). Skin temperature did not significantly differ between shifts (t = −0.92, *p* = 0.366, d = 0.41, 95% CI [−0.60, 0.23]). Night-shift workers had a significantly higher Epicore^®^ Activity Score (t = −3.06, *p* = 0.006, d = 1.37, 95% CI [−2.05, −0.39]). Total fluid intake (t = 1.83, *p* = 0.080, d = −0.79, 95% CI [−63.0, 1027.3]) and total sweat loss (t = −2.17, *p* = 0.062, d = 0.88, [−3885.8, 108.0]) were not significantly different between groups, but sweat sodium loss (t = −3.04, p = 0.006, d = 1.36, 95% CI [−4900.7, −919.4]) was significantly higher in night-shift workers than in morning-shift workers. Sweat rate did not differ significantly between groups (t = −1.79, *p* = 0.088, d = 0.80, 95% CI [−0.40, 0.03]). Full results, including means and standard deviations, are presented in Table 6.

**Table 6:** Comparison by shift schedule.

Variable	Shift	*n*	Mean ± SD	Min – Max
Shift duration	Morning	15	9.0 ± 0.5 h	7.7–9.6 h
	Night	9	9.4 ± 0.4 h	8.7–10.0 h
Activity level	Morning	14	32.3 ± 1.1	30.9–34.0
	Night	9	33.6 ± 0.6	32.9–34.8
Skin temperature	Morning	14	32.9 ± 0.5 °C	31.9–33.7 °C
	Night	9	33.1 ± 0.4 °C	32.4–33.5 °C
Total fluid intake	Morning	15	2,973 ± 723 g	1850–4,428 g
	Night	9	2,442 ± 408 g	1785–3,077 g
Sweat rate	Morning	14	0.34 ± 0.28 L/h	0.11–1.14 L/h
	Night	9	0.52 ± 0.17 L/h	0.31–0.79 L/h
Total sweat loss	Morning	14	3,014 ± 2,557 mL	1,020–10,144 mL
	Night	9	4,903 ± 1,624 mL	2,819–7,527 mL
Total sweat sodium loss	Morning	14	2,460 ± 2,294 mg	756–9,250 mg
	Night	9	5,370 ± 2,151 mg	2,410–8,488 mg

Data reported as mean ± standard deviation.

## 4 Discussion

To our knowledge, no studies to date have quantified acute and weekly markers of hydration, sweat profiles, and their potential associations with ergonomic injury risk in warehouse employees. Therefore, first, we assessed acute and weekly changes in pre- and post-shift USG and explored the association between change in urine color, total fluid intake, sweat loss, and wearable-derived ergonomic risk. Additionally, we explored the potential differences in these variables between morning and night-shift workers. We observed increases in USG from pre- to post-shift. We also observed associations between change in urine color and some, but not all, ergonomic outcomes. Finally, we observed significant differences in activity levels and sweat sodium losses by shift.

In partial agreement with our primary hypothesis, our results do show significant increases in USG from pre- to post-shift; however, we did not see significant increases in USG throughout the workweek. In our investigation, 80% of all USG samples were > 1.020, 39% of those were above 1.025, and 7% of those were > 1.030. These findings are comparable to results from previous research in underground miners (4) and agricultural workers (15), but much higher than the USG values seen in construction workers in the United Arab Emirates, among whom workers were, on average, adequately hydrated upon arriving at work (USG < 1.020) (36). When comparing pre- and post-shift proportions, 67% of all pre-shift USG samples were > 1.020, 29% of those were > 1.025, and 8% of those were > 1.030, while 81% of all post-shift USG samples were > 1.020, 53% of those were > 1.025, and 19% of those were > 1.030. This further aligns with the previous research in agricultural workers (15) and miners (4), showing a majority of workers arrived at work underhydrated and an even larger proportion left work underhydrated. This prevalence of underhydration suggests a strong need for hydration education and support for occupational workers given the potential risks of heat-related illness, reduced cognitive function and alertness (6, 7), motor control (8), and reduced physical work capacity and productivity (9, 10) that are associated with dehydration when working in hot and humid environments. We did not observe differences throughout the workweek. We expected that there would be a compounding effect of acute, insufficient hydration habits at work that would lead to higher urine concentrations over the course of the week. It is plausible that participants’ off-shift hydration habits compensated for the insufficient on-shift habits; however, this was not assessed in our investigation, and future work is needed.

In partial agreement with our secondary hypothesis, our results demonstrated significant associations between *Δ* urine color from pre- to post-shift and metrics of ergonomic risk. A one-unit change in Δ urine color was associated with a 12.6% higher total elevated risk movement count. After analyzing individual components of total elevated risk (high, moderate, mild-risk movements), we demonstrated that this association is predominantly in relation to mild-risk movements. A one-unit change in *Δ* urine color was associated with a 16.7% higher mild-risk movement count. While not statistically significant, we did see that the change in Δ urine color was associated with an 8.3 and 8.4% higher count of moderate- and high-risk movements, respectively. Although the full model, including total fluid intake, sweat rate, and urine color, was not significant, when individual predictors were investigated, we observed an association between higher changes in Δ urine color and higher injury risk scores. A one-unit change in Δ urine color was associated with a 2.7 higher injury risk score. When considering the measured range of injury risk scores in this investigation, this equates to an approximate 14% higher injury risk score. Contrary to our hypothesis, none of these ergonomic outcomes were significantly associated with total fluid intake or sweat rate. The significant association between increases in Δ urine color and mild ergonomic risk movements, but not moderate or high-risk movements, may be related to the movement patterns associated with each outcome (Figure 1). Previous laboratory research has shown an influence of hydration on peripheral motor control (8, 37) and a lack of association between postural outcomes and hydration (38). However, in opposition to this theory, other laboratory studies demonstrate that hydration is associated with posture and balance changes (39, 40). Given that mild-risk movements represent changes in elbow and shoulder movement angles, while moderate and high-risk movements reflect a combination of shoulder and back movement angles, we suggest future research should investigate to understand whether within-shift worsening of hydration status aligns more closely with upper-limb than postural movement variability in this setting. However, because this is the first investigation of its kind and the sample size is modest, more work is necessary to fully elucidate these potential relationships between hydration and ergonomic risk. Additionally, in contrast to our hypothesis, we did not see an association between total fluid intake and ergonomic risk. While some literature has suggested fluid intake is associated with these changes in motor control (41), which could impact ergonomic risk (42), our study did not see this association with practical, wearable-derived metrics of ergonomic risk in the field. This could be due to the large variability in sweat rate and activity level, yet modest variability in total fluid intake. However, without body weight, urine output, and food intake measurements, we could not estimate whole-shift fluid balance, which limits the interpretation of the null associations between fluid intake, sweat, and ergonomic outcomes.

In partial agreement with our hypothesis, there was a significant association between sweat rate and physical fatigue. Our findings suggest that a 1 L/h higher sweat rate was associated with a 26% higher change in self-reported fatigue from pre- to post-shift. However, we did not find evidence of an association between self-reported fatigue and *Δ* urine color or total fluid intake. These findings warrant future work to confirm if larger changes in self-reported fatigue from pre- to post-shift are more closely associated with the individual’s sweat response to the activity and heat stress experienced than hydration habits and status. Additionally, we did not find evidence of an association with physical energy, mental energy, or mental fatigue. It is not unexpected that associations were not seen between self-reported physical energy, mental energy, and mental fatigue. Measures of mental energy and fatigue were expected to be attenuated due to the complex, subjective relationship between uncontrolled psychological and environmental factors that are separate from the physiological factors measured in this study. Additionally, while energy and fatigue are related, they are distinct subjective states, allowing individuals to simultaneously experience varying levels of both (43, 44). To avoid overfitting our statistical models, given our sample size, these measurements of fatigue and energy were not included in our assessment of ergonomic risk; however, previous work has shown self-reported energy and fatigue may be associated with changes in gait (45–47), balance (45), and postural control (46). The combined findings of this investigation and previous work warrant future investigation of this association between fatigue, energy, and ergonomic risk in industrial athletes.

In our exploration of shift differences in sweat, hydration, and exertion, we saw night-shift workers averaged significantly higher activity scores and total sweat sodium loss compared to the morning-shift workers, despite similar shift durations. Although total fluid intake, sweat loss volume, and sweat rate were not significantly different by shift, there were numerically practical differences worth considering. For example, night-shift workers consumed 18% lower total fluid intake, even though the night-shift workers experienced ~63% higher total sweat losses (~53% higher sweat rate) and 118% higher total sweat sodium losses compared to the morning-shift workers. Even though BMI, age, and shift duration did not significantly differ between the two shift groups, it is worth noting that all three female subjects in this investigation worked the morning shift and had lower sweat losses (1,604 mL), sweat rates (0.20 L/h), and sweat sodium losses (1,511 mg). Additionally, the differences in activity and sweat variables could in part be described by the differences in the occupational responsibilities between the two shift groups. The site managers highlighted that morning-shift workers are often moving less frequently but lifting lower volumes of larger items, while night-shift employees are often moving more frequently but lifting larger volumes of smaller items. These findings provide support that there may be unique and individualized hydration needs even within sub-populations of industrial athletes, based on their specific occupational roles and responsibilities. However, future research is still needed in a larger sample across multiple warehouses to understand if these differences exist more broadly. Regardless of group, we see that these workers exhibit significant variability in their sweat and sweat sodium losses. There were losses as low as 1 liter per shift and as high as 10 liters per shift. There were also total sweat sodium losses as low as 756 mg and as high as 9,250 mg. These sweat profiles are unique compared to typical athletes who tend to have higher sweat rates over the course of a 2–3-h training session (5). Even though these workers may have moderate to low sweat rates compared to athletes, it is important to consider that this can lead to large sweat losses over a 9-h work shift. Based on our findings, we see these workers fall into a similar range of sweat rates and sweat sodium losses compared with those reported in other occupational populations (3, 4). Additionally, we observed similar fluid intake values to previous research on hydration status in construction workers (36).

This study provides novel insight into hydration and ergonomics in an understudied population of industrial athletes working in warehouses. However, it is not without its limitations. Because the power analysis was based on the primary aim, the study may have been potentially underpowered for the exploratory secondary and tertiary aims. Given the exploratory and observational nature of this investigation, ergonomic risk and shift-specific differences should be interpreted as associations rather than causal relationships. While USG is widely used as a practical marker of hydration status, it does not provide real-time fluid balance but rather reflects renal processing and may lag behind changes in total body water. It is also considered highly sensitive to the consumption of a large bolus of fluid (48) or food intake (49) and can diverge from changes in plasma osmolality (50); therefore, it may not be a stable marker reflective of whole-body hydration. Additionally, due to the limited time allotted for our team to collect measures and our efforts to reduce participant and site burden, we chose not to collect body weight, urine output, and food intake; therefore, fluid balance could not be calculated. Future work should focus on gathering fluid balance outcomes to better understand associations between fluid intake, sweat rate, dehydration, ergonomic risk, and fatigue. Although the LifeBooster Senz system derives its risk categories from established ergonomic standards, it is worth acknowledging that these outcomes are calculated using a proprietary algorithm, and there is currently no research validating the system against reference methods of ergonomic outcomes. While our investigation prioritized the inclusion of both female and male workers, due to the limited female population within the facility, sex-based comparisons were not feasible. Future work should investigate potential sex-based differences in occupational demands and hydration needs among warehouse workers.

## 5 Conclusion

In conclusion, most workers arrived at and left work underhydrated. We also found that these workers were exposed to hot and humid environments for ~9 h while engaging in moderate- to high-level activity and produced large quantities of sweat, with substantial sodium losses through sweat. Additionally, urine color and sweat rate may be associated with on-shift ergonomic risk and fatigue, respectively. Environmental health and safety (EHS) teams may use these preliminary findings to support improved hydration education that is individualized to the specific needs of warehouse workers. Additional research in this population is needed to fully elucidate the unique physiological demands, risks, and hydration needs of warehouse workers.

## 6 Practical applications

Environmental Health and Safety (EHS) teams may consider implementing optional urine-color education and urine color charts as inexpensive and easy-to-interpret hydration tools. For example, workers could be encouraged to aim for lighter-colored urine at the start of the shift and to limit darkening across the shift while maintaining practical access to fluids. Additionally, workers in this study exhibited a wide range of total sweat losses (~1–10 L/shift) and sweat sodium losses (756–9,250 mg), suggesting that even moderate sweat rates can translate into meaningful total fluid and electrolyte needs over a full 9-h workday. Practical on-site strategies include ensuring that sodium-containing fluids or foods are readily accessible for workers with high sweat sodium losses, incorporating brief opportunities to drink into the workflow, and reinforcing that hydration needs may vary substantially between employees due to differences in task demands and individual physiology. The association between sweat rate and self-reported changes in physical fatigue may be useful for supervisors and EHS teams to consider during long warm shifts to encourage workers to respond early to increasing fatigue with cooling opportunities, hydration access, and brief recovery breaks. Finally, the differences observed between morning- and night-shift workers (e.g., activity and sweat sodium loss) support the potential for individually tailored hydration strategies.

## Data Availability

The raw data supporting the conclusions of this article will be made available by the authors, without undue reservation.
